# Immunomodulatory asthma therapy in the equine animal model: A dose‐response study and evaluation of a long‐term effect

**DOI:** 10.1002/iid3.252

**Published:** 2019-05-29

**Authors:** John Klier, Carolin Bartl, Sabine Geuder, Katharina J. Geh, Sven Reese, Lutz S. Goehring, Gerhard Winter, Heidrun Gehlen

**Affiliations:** ^1^ Centre for Clinical Veterinary Medicine, Equine Clinic Ludwig‐Maximilians‐University Munich Germany; ^2^ Department of Veterinary Medicine, Equine Clinic, Surgery and Radiology Free University of Berlin Berlin Germany; ^3^ Department of Pharmacy, Pharmaceutical Technology and Biopharmaceutics Ludwig‐Maximilians‐University Munich Germany; ^4^ Department of Veterinary Medicine, Equine Clinic, Surgery and Radiology Free University of Berlin Berlin Germany

**Keywords:** allergic asthma, extrinsic asthma, heaves, inhalation, neutrophilic asthma

## Abstract

**Introduction:**

Equine asthma represents a naturally occurring animal model for human allergic neutrophilic asthma. Inhalative nanoparticle‐bound cytosine‐phosphate‐guanosine (CpG‐GNP) immunotherapy, independent of specific allergens, has already shown promising clinical and immunological results in previous studies and offers the possibility to treat the underlying cause of the disease. This study analyses the relationship between dose and response, and evaluates a possible long‐term effect.

**Methods:**

In the prospective, randomised, double‐blind clinical field study, 29 horses suffering from equine asthma received 10 inhalation treatments with either 187.5 µg CpG‐GNP (CpG single dose [CpGsd]; n = 11), 375 µg CpG‐GNP double dose (CpG double dose [CpGdd]; n = 9) (q48h for 20 days) or 1600 µg beclomethasone (n = 9) (q24h for 10 days). Each horse was examined three times: before the treatment (I), immediately after the 10 inhalations (II), and 8 weeks after the final inhalation (III). The three groups were compared according to clinical and laboratory parameters. The study examined the sustainability of the long‐term effect of the treatment after 8 weeks, as well as the tolerability of the formula as a double dose.

**Results:**

The CpGsd resulted in a significant improvement in 82% of the parameters, the CpGdd in 72%. In the long‐term evaluation, the CpGsd showed a significant improvement in 100% of the parameters in comparison to the initial values, the CpGdd in 67%. On the immunological level, the bronchoalveolar lavage revealed a significant reduction of IL‐4, IL‐8, and interferon‐γ.

**Conclusion:**

Both CpG groups displayed significant improvements in clinical and laboratory parameters, especially regarding the long‐term effect of CpGsd. Doubling the CpG dose did not result in any improvement in comparison to the original single dose. On the immunological level, an anti‐inflammatory, as well as an immunomodulatory effect, apart from a Th2‐dominated immune response, could be observed. This immunomodulatory inhalation treatment could indicate a new possibility for human allergic asthma therapy.

**Abbreviations**
AaDO_2_: Alveolar‐arterial oxygen gradient
BAL: bronchoalveolar lavage
CpG‐ODN: Cytosine‐phosphate‐guanosine‐oligodeoxynucleotide
CpGsd: CpG single dose
CpGdd: CpG double dose
GNP: gelatine nanoparticle
HOARSI: horse owner assessed respiratory signs index
HPW: highly purified water
IBH: insect bite hypersensitivity
IFN: interferon
IL: interleukin
MACS: magnetic‐activated cell sorting
MHS: multiple hypersensitivities
PAMPS: pathogen‐associated molecular patterns
PPID: pituitary pars intermedia dysfunction
Th: T‐helper cell
TLR‐9: toll‐like receptor 9
Tregs: T regulatory cells

## 1 INTRODUCTION

Equine asthma (recurrent airway obstruction, heaves) is one of the most significant chronic inflammatory allergic diseases of the respiratory tract in horses, and can affect about 15% or even higher numbers of mature horses kept in stables.^1^, ^2^

Equine asthma refers to a multifactorial disease.^3^ The main causes are found in the management of the horses affected, which is why equine asthma may also be described as a disease of domestication.^4^

There are over 50 different types of allergens found in hay and straw dust.^5^ Mould spores, such as *Aspergillus fumigatus* or *Faenia rectivirgula*, have an especially high allergen potential. However, storage mites, such as *Lepidoglyphus destructor*, and bacterial endotoxins are also potential trigger factors. Further factors also contribute, such as inorganic dusts (eg, silicates), cold and dry air, as well as irritant gases (eg, ammonia).^5^, ^6^

Besides the environmental factors, a genetic predisposition also plays an important role.^7^ A polygenetic cause for the heredity of equine asthma is considered highly probable.^8^

The recurring restriction of the airways is caused on the one hand by the reversible pathomechanisms bronchospasm, dys‐ and hypercrinia and the migration of neutrophilic granulocytes, and on the other hand by the irreversible remodelling processes in the airways (“airway remodelling”) with smooth muscle proliferation.^6^ Exposure to allergens irritating the mucus membranes results in a constriction of the bronchi ca. 3 to 5 hours after exposure.^9^

The inflammation of the airways within the lumen is primarily due to neutrophilic granulocytes, while lymphocytes, mastocytes, plasma cells are located submucosally.^10^, ^11^ The neutrophils can release mediators, such as elastase, free oxygen radicals and leukotriene B4, which leads to tissue damage. In addition, the hypersecretion of the glands in the airways initiates an increased mucus production with an altered composition of the mucus.^6^

As a result, the reduction of the lung volume cannot be entirely reversed, even with the administration of bronchodilators.^6^, ^12^ Furthermore, it can result in both alveolar ventilation disorders as well as perfusion disorders, due to a ventilation/perfusion mismatch.^6^

The allergen‐independent immunotherapy is a promising treatment method, by which regulatory T cells (Treg) are activated and the correct T helper (Th) cell balance can be restored.^13^ Nanoparticulate cytosine‐phosphate‐guanosine oligodeoxynucleotides (CpG‐ODNs, CpG‐GNP), through pathogen‐associated molecular patterns recognized by toll‐like receptors, were used as immunomodulators in both human and equine asthma,^14^, ^15^, ^16^ and stimulate T helper subsets with a Th2/Th1 switch and activation of Treg.^17^ In equine lungs, macrophages, epithelial cells, capillary endothelial cells, and neutrophilic granulocytes were proven to be positive for TLR‐9.^18^

Gelatine nanoparticles (GNP) are biodegradable and immunologically inert, and are a safe and effective carrier system for the CpG inhalation.^19^, ^20^

The aims of this dose‐response study were to compare two different dosages of the CpG‐GNP inhalation therapy and to examine its clinical efficacy and to evaluate a possible long‐term effect over 8 weeks. The results were compared to a once daily corticosteroid inhalation treatment. The hypothesis was that the immunmodulatory CpG treatment has an ongoing long‐term effect over 8 weeks without further medication or management strategies in a natural environment.

## 2 METHODS

### 2.1 Study design and patients

In total, 29 horses with equine asthma participated in the prospective, randomized, double‐blind clinical field study. The mean age was 19 (±4.7) years old, 20 males and 9 females, with 11 different breeds, while 15 of the patients were held in open stables, 13 in stables that opened to the outside, and 1 in an entirely internal stable (bedding material: 18 horses with straw, 4 horses with wood shavings, 2 horses with sawdust, 4 horses without bedding material; food: 15 horses got dry hay, 11 horses soaked or steamed hay and 3 horses haylage). Horses were randomly distributed to the three groups according to age, housing (contact to hay and straw), and breed (ponies vs horses) to ensure uniform distribution, beclomethason group: 17.9 (±4.0) years; CpG single dose (CpGsd) group: 20.1 (±3.0) years; and CpG double dose (CpGdd) group: 19.0 (±6.8) years). No provocation via contact to hay and straw was performed before or during the study. Familiar housing was not changed during the entire study.

The study was approved by the regional legal agency for animal experiments of the Government of Upper Bavaria, Germany (No. 55.2‐1‐54‐2532‐207‐13). All of the horse owners signed an informed consent. The study was performed in accordance to the guidelines for animal studies (ARRIVE) and clinical trials (CONSORT).

### 2.2 Inclusion criteria

The following inclusion criteria were chosen: upon contact to dusty hay and straw increased respiratory rate at rest (>16/minute); an increased abdominal breathing effort; pathological findings in the lung auscultation; increased percentages of neutrophilic granulocytes in the airways via bronchoalveolar lavage (BAL) (>25%); reduced arterial blood gas levels at rest (partial oxygen pressure <90 mmHg); increased interpleural pressure (>15 cmH_2_O); clinical symptoms triggered by contact to hay and straw; and reduction of the symptoms upon keeping the horses outside.^6^ Due to the heterogeneous group of horses (different barns, different stages of disease and duration of disease) and the character of the study as a field study the starting point of all horses could not be exactly the same. Other causes of respiratory disease were ruled out via the lack of signs of infection and fever, chronicity, reversibility of signs via avoidance of contact to trigger factors. Initially 36 horses were included into the study. Five horses had to be excluded at the beginning because they were in remission and two dropped out during the study due to the administration of medications (nonsteroidal anti‐inflammatory drugs [NSAID] or corticosteroids) unrelated to the study to treat lameness causing the uneven distribution of the sample size of the three treatment groups CpGsd (n = 11), CpGdd (n = 9), and beclomethasone (n = 9). All of the horses remained in their own stables during the entire study, and continued to receive their familiar management and feed. There were no attempts to improve the stabling or management of the patients during this time. The horses of all three groups were treated in parallel, to limit the effects of seasonal or weather‐related influencing factors on the results. The study was performed between April and August. None of the horses received additional medication within 8 weeks previous to the study or during the study itself.

### 2.3 Intervention

GNPs were produced and loaded with CpG‐ODN 2216 (Biomers GmbH, Ulm, Germany) as described previously.^15^, ^16^, ^21^ For the CpG‐GNP single dose, each inhalation utilized 187.5 μg CpG‐ODN, bound to 3.75 mg GNP and dispersed in 2.5 mL highly purified water (HPW), with a final concentration of 1.5 mg/mL GNP and 0.075 mg/mL CpG‐ODN as described previously.^15^, ^16^, ^22^ For the CpG‐GNP double dose, each inhalation utilized 375 μg CpG‐ODN, bound to 7.5 mg GNP and dispersed in 5 mL HPW with a final concentration of 1.5 mg/mL GNP and 0.075 mg/mL CpG‐ODN. All inhalation treatments (CpG and beclomethasone) were administered via the Equine Haler (Equine HealthCare Aps, Hoersholm, Denmark) combined with the Aeroneb Go micropump nebulizer (Aerogen, Galway, Ireland) as described previously.^15^, ^16^

The 10 CpG‐GNP inhalations were administered to each horse every 2 days, in accordance with the protocol from the preceding studies for altogether 20 days. The 48 hour interval between inhalations was chosen according to the in vivo stability of CpG‐ODN shown to be 48 hours^23^ and the reliable protocol.^15^, ^16^ The beclomethasone group (Sanasthmax 400 Mikrogramm/ 1 mL, Chiesi GmbH, Hamburg) received once daily inhalations (every 24 hours) of 1600 µg (4 mL), on 10 consecutive days.^24^ The q24h interval for beclomethasone treatment was chosen instead of a q12h interval because of no feasibility of this field study. The inhalations were administered by two people (CB, SG). Both clinical and laboratory examinations were blind.

### 2.4 Clinical and laboratory evaluations

The first examination of the horses was completed before treatment (I) and included a thorough clinical examination (auscultation, respiratory rate and type, nasal discharge, provocation test, and lung percussion), an evaluation of the pulmonary function parameters (interpleural pressure, arterial blood gas values), a bronchoscopy with evaluation of the amount of mucus in the airways, as well as a BAL. In addition, immunological parameters (detailed below) were determined from the BAL. The second examination (II) was conducted directly after the 10 inhalations, either after 3 weeks (for the CpG‐GNP treatment) or after 10 days (for the beclomethasone treatment). The third examination (III) was conducted 8 weeks after the last inhalation treatment.

The clinical examination was conducted as described previously^15^, ^16^, ^22^ and in accordance with established and standardized scoring systems^5^, ^25^, ^26^ (Supporting Information).

All of the horses were observed clinically on a daily base by the owners and CB and SG during inhalation treatment, and completely with endoscopy, interpleural pressure measurement, blood gas analysis, and blood work during the three examination points to determine the tolerability of the dosage and the treatment regimen. This observation recorded any nasal discharge (consistency and quality), cough (at rest and during exertion), signs of bronchospasm (wheezing and whistling, nasal flaring, and increased abdominal breathing effort), increased respiratory rate at rest, visible redness or swelling of the mucous membranes within the scope of the endoscopic examination, follicular hyperplasia, and/or decreased blood gas values. In addition, the rectal temperature was measured daily and a white blood cell count, a differential blood count and a fibrinogen test were evaluated within the scope of the three control examinations for signs of adverse systemic effects.

BAL was performed with 250 mL of sterile saline. BAL was centrifuged (300 g for 6 minutes) and the supernatant was immediately frozen with liquid nitrogen and stored at −80°C until the samples were evaluated. The cell suspension was placed on ice during transportation.

### 2.5 Cytokine assay

The analysis of the cytokines IL‐4, IL‐10, IL‐17, and interferon‐γ from BAL supernatant was conducted in the Cornell Animal Health Diagnostic Center (Cornell University, Ithaca, NY) via the Multiplex Assay^27^ (Supporting Information). For this purpose, the samples were sent frozen on dry ice (TNT, Germany).

### 2.6 Magnetic‐activated cell sorting

The BAL cell suspensions were stored on ice immediately after extraction and were processed in the laboratory within a maximum of 3 hours.

Following this, the samples were centrifuged for 10 minutes at 300 g.

To free the samples from mucus as much as possible, the BAL was filtered through a single layer of sterile gauze. Subsequently, 50 µL of the cell suspension was extracted and mixed with 50 µL of trypan blue (live/dead staining) and the percentage of living cells were determined under the microscope.^28^

The positive selection of CD4^+^ T lymphocytes occurred in accordance with the magnetic‐activated cell sorting (MACS)‐ protocols of the company Miltenyi Biotec.^29^, ^30^

The purity of the CD4^+^ cells was monitored via flow cytometry (FACS‐Gerät: MACSQuant VYB; Miltenyi Biotec GmbH, Bergisch Gladbach, Germany) following the MACS separation (Figures S4‐S6). These checks were carried out randomly and were already tested in the preliminary stages of the execution of the study. For this purpose, a fluorescein isothiocyanate (FITC) staining dye was utilized, coupled to a CD4^+^ antibody (FITC‐conjugated Goat antimouse IgG antibody; Miltenyi Biotec GmbH), to test the purity of the fractionation.^28^ In this way, the successful selection of CD4^+^ could be determined at 98% (Figure S6).

### 2.7 Messenger RNA assay

The quantitative analysis of the messenger RNA (mRNA) from the CD4^+^ lymphocytes selected via MACS was conducted in the Gluck Center, KY via Real Time PCR. To this end, the samples were sent via a cooled transport (Medpak Thermo, TNT, Troisdorf).

This analysis determined the mRNA of the transcription factors Forkhead Box Protein P3 (FoxP3), T‐bet (Th‐1‐specific T‐box transcription factor), GATA‐3 (Trans‐acting T‐cell‐specific transcription factor) and the cytokines IL‐8 and TGF‐β (transforming growth factor‐β).^30^, ^31^, ^32^

The isolation of the mRNA was conducted according to a fixed schema (Supporting Information).^33^ The results were mathematically adjusted with the help of a reference gene (BGus).^30^ For the final results, the logarithmic value of relative quantification was determined for each of the parameters examined.

### 2.8 Statistical analysis

The statistical analysis of the results utilized the GraphPad Prism 5 Software (Graphpad Software Inc., La Jolla, CA). The follow‐up examinations within the treatment groups (between the first and second, and between the first and third examinations) utilised the Wilcoxon matched‐pairs test for nonparametric data. The comparison between the groups was conducted with the Mann Whitney Test (beclomethasone with CpGsd or CpGdd) with adjustment according to Bonferroni for multiple tests. Both were performed one‐tailed. In addition, the effect size (Cohen's *d*) of each treatment on the parameters examined was determined (large clinical effect: *d* > 0.8; medium effect: 0.5‐0.8; small effect: 0.2‐0.5). The calculation of the effect size was performed using the EffectSize Calculator (http://davidmlane.com/hyperstat/effect_size.htm, Robert Coe).^34^ Sample size calculation was performed with 12 subjects per group (one‐tailed, Cohen's *d* > 1 for relevant clinical effect with a power of >0.8 and α *P* = 0.05).

## 3 RESULTS

### 3.1 Adverse effects

During the entire study, no clinical or clinical‐pathological indications of local or systemic side effects could be determined as the result of any of the inhalation treatment. Two horses dropped out during the study due to the administration of medications (NSAID or corticosteroids) unrelated to the study to treat lameness.

### 3.2 Respiratory rate at rest

**Figure 1:**
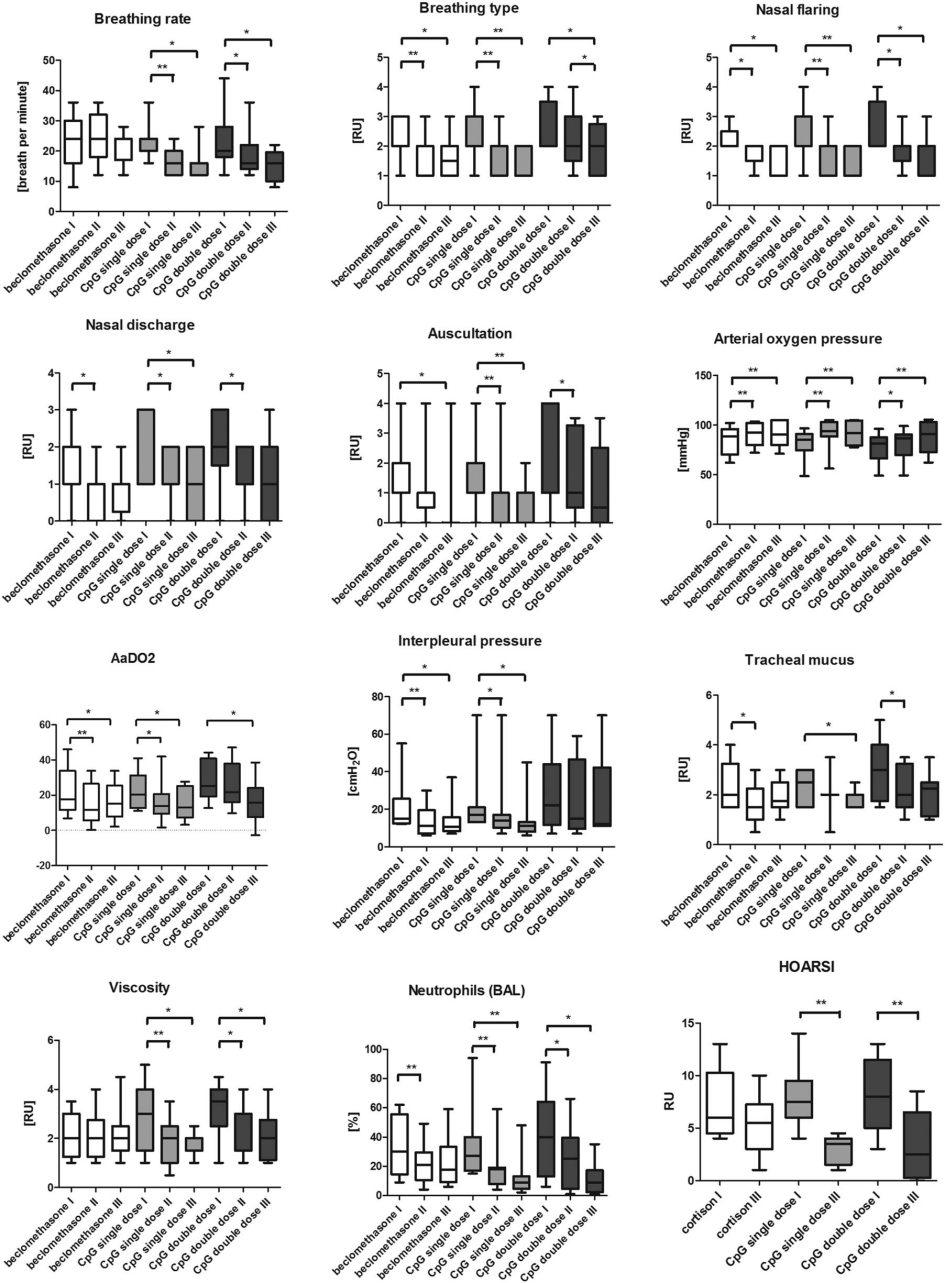
Absolute values of breathing rate (n/minute) and pattern (RU), lung auscultation (RU), interpleural pressure measurement (cmH_2_O), oxygen partial pressure (mmHg), AaDO_2_ (mmHg), percentage of neutrophils in BAL (%), nasal discharge (RU), endoscopic quantification of secretion (RU) and viscosity (RU) in equine asthma‐affected horses at first examination before inhalation treatment (I), after 10 inhalations (II) of beclomethasone (n = 9 equine asthma‐affected horses), CpG single dose (CpGsd) group (n = 11 equine asthma‐affected horses) and CpG double dose (CpGdd) group (n = 9 equine asthma‐affected horses) and after 8 weeks without any treatment to evaluate long‐term effect (III). Calculated value of *P* < 0.05 displayed as *, *P* < 0.01 as **. AaDO_2_, alveolar‐arterial oxygen gradient; BAL, bronchoalveolar lavage; RU, relative unit

Only the two CpG groups showed a significant improvement in the respiratory rate directly after treatment (Table 1; Figure 1). This effect was also still evident after 8 weeks. The beclomethasone group did not show any improvement.

**Table 1:** Depicted are changes of clinical [%] and laboratory parameters [% or lnRQ] of all three therapy groups (Beclomethasone n = 9, CpG single dose n = 11, CpG double dose n = 9), between first and second (I–II) and first and third examination (I–III), mean (±SD), logarithmic value of relative quantification (lnRQ), relative unit (RU; cf. Suppl.), p‐value and effect size Cohen´s d (large clinical effect: d > 0.8; medium effect: 0.5–0.8; small effect: 0.2–0.5)

	Breathing rate (n/min)		Breathing type (RU)		Nasal flaring (RU)	
	I‐II	I‐III	I‐II	I‐III	I‐II	I‐III
Beclomethasone	+20.8% (±54.6) *P* = 0.219 *d* = −0.199	+2.7% (±26.4) *P* = 0.408 *d* = 0.161	−27.8% (±2.0) *P* = 0.010 *d* = 0.845	−37.5% (±7.3) *P* = 0.011 *d* = 0.967	−18.5% (±22.7) *P* = 0.036 *d* = 0.909	−39.6% (±17.7) *P* = 0.011 *d* = 1.670
CpG single dose	−24.4% (±21.7) *P* = 0.007 *d* = 1.256	−26.0% (±6.7) *P* = 0.044 *d* = 1.334	−32.6% (±23.7) *P* = 0.004 *d* = 1.196	−40.5% (±22.4) *P* = 0.006 *d* = 1.670	−38.6% (±21.8) *P* = 0.002 *d* = 1.229	−40.9% (±21.6) *P* = 0.006 *d* = 1.538
CpG double dose	−15.6% (±24.1) *P* = 0.024 *d* = 0.535	−30.8% (±3.8) *P* = 0.022 *d* = 1.061	−15.7% (±31.9) *P* = 0.065 *d* = 0.293	−31.3% (±23.9) *P* = 0.036 *d* = 0.471	−26.8% (±21.0) *P* = 0.013 *d* = 1.046	−34.4% (±24.6) *P* = 0.031 *d* = 1.107

	Nasal discharge (RU)		Auscultation (RU)		Arterial oxygen pressure (mmHg)	
	I‐II	I‐III	I‐II	I‐III	I‐II	I‐III
Beclomethasone	−29.6% (±80.3) *P* = 0.049 *d* = 1.023	−33.3% (±38.8) *P* = 0.095 *d* = 0.901	−22.2% (±61.8) *P* = 0.117 *d* = 0.261	−87.5% (±35.4) *P* = 0.031 *d* = 0.710	+7.7% (±7.2) *P* = 0.004 *d* = 0.438	+10.6% (±6.9) *P* = 0.008 *d* = 0.514
CpG single dose	−33.3% (±53.8) *P* = 0.010 *d* = 1.243	−54.5% (±62.0) *P* = 0.012 *d* = 1.787	−52.3% (±48.0) *P* = 0.005 *d* = 0.449	−65.9% (±39.2) *P* = 0.007 *d* = 0.973	+10.6% (±9.9) *P* = 0.005 *d* = 0.555	+15.4% (±17.9) *P* = 0.003 *d* = 0.856
CpG double dose	−14.8% (±68.4) *P* = 0.048 *d* = 0.759	−31.3% (±00.2) *P* = 0.145 *d* = 0.909	−25.0% (±37.0) *P* = 0.028 *d* = 0.385	−42.2% (±48.6) *P* = 0.055 *d* = 0.702	+4.3% (±6.1) *P* = 0.049 *d* = 0.221	+14.6% (±9.7) *P* = 0.008 *d* = 0.697

	AaDO_2_ (mmHg)		Interpleural pressure (cmH_2_O)		Tracheal mucus (RU)	
	I‐II	I‐III	I‐II	I‐III	I‐II	I‐III
Beclomethasone	−41.0% ( ±26.5) *P* = 0.002 *d* = 0.549	−24.4% (28.7) *P* = 0.023 *d* = 0.441	−30.0% (±21.1) *P* = 0.008 *d* = 0.581	−30.3% (±25.9) *P* = 0.034 *d* = 0.532	−26.6% (±36.6) *P* = 0.027 *d* = 0.677	−16.1% (±14.1) *P* = 0.053 *d* = 0.483
CpG single dose	−27.7% (±41.1) *P* = 0.013 *d* = 0.562	−30.1% (±53.2) *P* = 0.032 *d* = 0.829	−20.3% (±27.3) *P* = 0.021 *d* = 0.251	−29.7% (±48.0) *P* = 0.036 *d* = 0.551	−11.4% (±41.1) *P* = 0.110 *d* = 0.402	−18.3% (±27.5) *P* = 0.049 *d* = 0.964
CpG double dose	−12.6% (±22.6) *P* = 0.065 *d* = 0.271	−49.0% (±37.9) *P* = 0.016 *d* = 1.030	−6.8% (±42.9) *P* = 0.116 *d* = 0.166	−4.9% (±54.2) *P* = 0.326 *d* = 0.186	−18.8% (±24.1) *P* = 0.029 *d* = 0.660	−28.2% (±30.6) *P* = 0.073 *d* = 0.841

	Viscosity (RU)		Neutrophils (%)		HOARSI (RU)
	I‐II	I‐III	I‐II	I‐III	I‐III
Beclomethason**e**	+12.7% (±74.0) *P* = 0.465 *d* = 0.000	+4.8% (±53.6) *P* = 0.462 *d* = −0.200	−31.8% (±31.0) *P* = 0.010 *d* = 0.669	−11.4% (±92.4) *P* = 0.080 *d* = 0.555	−20.0% (±40.9) *P* = 0.068 *d* = 0.589
CpG single dose	−28.3% (±39.4) *P* = 0.008 *d* = 0.896	−19.0% (±66.8) *P* = 0.036 *d* = 1.230	−43.3% (±21.4) *P* = 0.002 *d* = 0.715	−61.3% (±26.0) *P* = 0.002 *d* = 1.064	−56.5% (±20.3) *P* = 0.004 *d* = 2.135
CpG double dose	−22.3% (±18.5) *P* = 0.011 *d* = 0.765	−28.1% (±41.1) *P* = 0.041 *d* = 0.765	−33.9% (±46.9) *P* = 0.029 *d* = 0.568	−62.2% (±31.4) *P* = 0.016 *d* = 1.232	−56.2% (±26.2) *P* = 0.009 *d* = 1.189

	IL‐4 (pg/mL) BAL		IL‐10 (pg/mL) BAL		IL‐17 (U/mL) BAL	
	I‐II	I‐III	I‐II	I‐III	I‐II	I‐III
Beclomethasone	−23.8% *P* = 0.037 *d* = 0.487	−29.8% *P* = 0.012 *d* = 0.615	+26.7% *P* = 0.125 *d* = −0.293	+24.6% *P* = 0.500 *d* = −0.238	+50% *P* = 0.028 *d* = −0.439	+33.9% *P* = 0.500 *d* = −0.214
CpG single dose	−10.6% *P* = 0.004 *d* = 0.143	−10.9% *P* = 0.004 *d* = 0.147	−42.9% *P* = 0.116 *d* = 0.526	+15.7% *P* = 0.216 *d* = −0.054	−31.3% *P* = 0.086 *d* = 0.260	−67.7% *P* = 0.062 *d* = 0.683
CpG double dose	−5.6% *P* = 0.313 *d* = 0.035	6.4% *P* = 0.500 *d* = ‐0.044	−34.2% *P* = 0.320 *d* = 0.305	+104.1% *P* = 0.156 *d* = −0.520	−1.7% *P* = 0.500 *d* = 0.012	−70.2% *P* = 0.146 *d* = 0.780

	IFN‐γ (U/mL) BAL		FoxP3 (lnRQ) CD4 cells BAL		TGF‐β (lnRQ) CD4 cells BAL	
	I‐II	I‐III	I‐II	I‐III	I‐II	I‐III
Beclomethasone	−8.8% *P* = 0.101 *d* = 0.773	0% *P* = 0.412 *d* = 0.000	+0.3 lnRQ *P* = 0.277 *d* = −0.410	+0.9 lnRQ *P* = 0.023 *d* = −1.317	−4.2 lnRQ *P* = 0.180 *d* = 0.198	+0.1 lnRQ *P* = 0.148 *d* = −0.013
CpG single dose	−7.8% *P* = 0.021 *d* = 0.571	−3.9% *P* = 0.163 *d* = 0.363	−0.4 lnRQ *P* = 0.138 *d* = 0.618	−1.1 lnRQ *P* = 0.032 d = 1.013	−0.3 lnRQ *P* = 0.120 *d* = 0.444	−0.1 lnRQ *P* = 0.382 *d* = 0.036
CpG double dose	−5.4% *P* = 0.195 *d* = 0.332	−5.4% *P* = 0.392 *d* = 0.362	−0.3 lnRQ *P* = 0.273 *d* = 0.192	−0.4 lnRQ *P* = 0.188 *d* = 0.314	−2.4 lnRQ *P* = 0.213 *d* = 0.529	−0.1 lnRQ *P* = 0.320 *d* = 0.305

	T‐BET (lnRQ) CD4 cells BAL		GATA‐3 (lnRQ) CD4 cells BAL		IL‐8 (lnRQ) CD4 cells BAL	
	I‐II	I‐III	I‐II	I‐III	I‐II	I‐III
Beclomethasone	+0.5 lnRQ *P* = 0.422 *d* = −0.376	+0.6 lnRQ *P* = 0.078 *d* = −0.472	+0.3 lnRQ *P* = 0.326 *d* = −0.338	+0.4 lnRQ *P* = 0.039 *d* = −0.545	0 lnRQ *P* = 0.410 *d* = 0.062	−0.2 lnRQ *P* = 0.344 *d* = 0.212
CpG single dose	+0.3 lnRQ *P* = 0.065 *d* = 0.464	−1.4 lnRQ *P* = 0.034 *d* = 1.056	−0.8 lnRQ *P* = 0.062 *d* = 0.889	−1.5 lnRQ *P* = 0.051 *d* = −0.790	−0.5 lnRQ *P* = 0.160 *d* = 0.369	−0.9 lnRQ *P* = 0.016 *d* = 0.690
CpG double dose	‐0.4 lnRQ *P* = 0.213 *d* = 0.316	‐0.4 lnRQ *P* = 0.191 *d* = 0.326	‐0.2 lnRQ *P* = 0.410 *d* = 0.267	‐0.7 lnRQ *P* = 0.104 *d* = ‐0.746	‐0.3 lnRQ *P* = 0.410 *d* = 0.213	−1.1 lnRQ *P* = 0.020 *d* = 0.990

Abbreviations: AaDO_2_, alveolar‐arterial oxygen gradient; BAL, bronchoalveolar lavage; CpG, cytosine‐phosphate‐guanosine; CpGsd, CpG single dose; IFN, interferon; IL, interleukin; lnRQ, logarithmic value of relative quantification; RU, relative unit.

In the comparison of the three treatment groups, the CpGsd could significantly improve the respiratory rate directly after treatment, in comparison to the beclomethasone therapy (Table S3). A significant improvement in the respiration rate was also determined after 8 weeks, both in the CpGsd and in the CpGdd, in comparison to the beclomethasone group (Table S3).

There was no significant difference determined between the two CpG treatment groups, neither between I and II, nor regarding the long‐term effect (III).

The comparison between the group of all horses treated with CpG and the beclomethasone group revealed a significant difference on the side of the CpG therapy, both between I and II, as well as between I and III (Table 2).

**Table 2:** Comparison in percent of all CpG treated horses (single and double dose; n = 20) with beclomethasone treatment (n = 9) between first and second (I‐II) and first and third examination (I‐III)

	Treatments in comparison	Examination stage I‐II	Examination stage I‐III
Breathing rate	Both CpG groups—beclomethasone group	*P* = 0.006	*P* = 0.004
Breathing type	Both CpG groups—beclomethasone group	*P* = 0.903	*P* = 0.956
Nasal flaring	Both CpG groups—beclomethasone group	*P* = 0.216	*P* = 0.446
Nasal discharge	Both CpG groups—beclomethasone group	*P* = 0.458	*P* = 0.190
Auscultation	Both CpG groups—beclomethasone group	*P* = 0.237	*P* = 0.034
Arterial oxygen pressure	Both CpG groups—beclomethasone group	*P* = 0.453	*P* = 0.190
AaDO_2_	Both CpG groups—beclomethasone group	*P* = 0.057	*P* = 0.042
Interpleural pressure	Both CpG groups—beclomethasone group	*P* = 0.097	*P* = 0.447
Tracheal mucus	Both CpG groups—beclomethasone group	*P* = 0.500	*P* = 0.106
Viscosity	Both CpG groups—beclomethasone group	*P* = 0.017	*P* = 0.021
Neutrophils	Both CpG groups – beclomethasone group	*P* = 0.327	*P* = 0.021
HOARSI	Both CpG groups—beclomethasone group	_	*P* = 0.007
IL‐4 BAL	Both CpG groups—beclomethasone group	*P* = 0.190	*P* = 0.029
IL‐10 BAL	Both CpG groups—beclomethasone group	*P* = 0.167	*P* = 0.237
IL‐17 BAL	Both CpG groups— beclomethasone group	*P* = 0.089	*P* = 0.174
IFN‐γ BAL	Both CpG groups—beclomethasone group	*P* = 0.491	*P* = 0.343
FoxP3 CD4 BAL	Both CpG groups—beclomethasone group	*P* > 0.999	*P* = 0.257
TGF‐ β CD4 BAL	Both CpG groups—beclomethasone group	*P* = 0.491	*P* = 0.092
T‐BET CD4 BAL	Both CpG groups—beclomethasone group	*P* > 0.999	*P* > 0.999
GATA‐3 CD4 BAL	Both CpG groups—beclomethasone group	*P* > 0.999	*P* > 0.999
IL‐8 CD4 BAL	Both CpG groups—beclomethasone group	*P* > 0.999	*P* = 0.703

Abbreviations: AaDO_2_, alveolar‐arterial oxygen gradient; BAL, bronchoalveolar lavage; CpG, cytosine‐phosphate‐guanosine; HOARSI, horse owner assessed respiratory signs index; IL, interleukin.

### 3.3 Breathing type

All three therapy regimens resulted in a significant improvement in the breathing type both directly after treatment, as well as after 8 weeks (Table 1; Figure 1). The CpGdd showed the smallest effect (Table 1).

The comparison of the three treatment groups determined no significant differences (Table S3).

In the comparison between all horses treated with CpG and the beclomethasone group, there were no significant differences observed between either I and II, or between I and III (Table 2).

### 3.4 Nasal flaring

All three treatment groups showed a significant clinical improvement of nasal flaring, both directly after the treatment, as well as after 8 weeks (Table 1; Figure 1). The greatest effects were evident after 8 weeks (Table 1).

The comparison between the three forms of treatment determined no significant differences (Table S3).

In the comparison between all horses treated with CpG and the beclomethasone group, there were no significant differences observed between either I and II, or between I and III (Table 2).

### 3.5 Nasal discharge

All three treatment regimens demonstrated a marked reduction of nasal discharge both directly after treatment and after 8 weeks (Table 1; Figure 1). Directly after the treatment, the CpGsd and beclomethasone groups showed significantly greater effects than the CpGdd (Table 1). After 8 weeks, the greatest effects and clinical improvements were demonstrated by the CpGsd (Table 1).

However, no significant differences were determined in the comparison of the different forms of therapy (Table S3).

In the comparison of all horses treated with CpG with the beclomethasone group, there were no significant differences observed between either I and II, or I and III (Table 2).

### 3.6 Auscultation of the airways

Both CpG groups showed a significant improvement in the auscultation findings after treatment (Table 1; Figure 1). After 8 weeks also the beclomethasone group demonstrated a significant improvement (Table 1).

The comparison of the three treatment forms revealed no significant difference, except between the CpGdd and the beclomethasone group after 8 weeks (Table S6).

In the comparison of the long‐term effect of treatment between all horses treated with CpG and the beclomethasone group, a significant improvement in the auscultation findings was determined in the horses receiving CpG therapy (Table 2).

### 3.7 Partial pressure of oxygen

All three groups showed a significant improvement in the partial pressure of oxygen both directly after treatment as well as after 8 weeks (Table 1; Figure 1). There was no significant difference between the treatment groups (Table S3).

The comparison between all horses treated with CpG with the beclomethasone group indicated no significant difference between I and II or between I and III (Table 2).

### 3.8 Alveolar‐arterial oxygen gradient

The beclomethasone and CpGsd group showed a significant improvement in the alveolar‐arterial oxygen gradient (AaDO_2_) directly after treatment, as well as a sustained effect over 8 weeks including the CpGdd group (Table 1; Figure 1). In the CpG groups, however, this was a large effect compared to the beclomethasone group (Table 1).

Between the treatment regimens, no significant difference was determined (Table S3), except between the beclomethasone group and the CpGdd between I and II in favour of the beclomethasone group.

The comparison of all horses treated with CpG with the beclomethasone group showed a significant long‐term effect over 8 weeks with the CpG therapy (Table 2).

### 3.9 Measurement of interpleural pressure

Both the beclomethasone group as well as the CpGsd group produced a significant reduction of the interpleural pressure directly after treatment and after 8 weeks (Table 1; Figure 1).

The comparison of treatments indicated no significant advantages to any one treatment form (Table S3).

The comparison of all horses treated with CpG with the beclomethasone group indicated no significant differences between I and II or between I and III (Table 2).

### 3.10 Amount of tracheal secretion

All three treatment regimens resulted in a significant reduction of the amount of tracheal secretion (Table 1; Figure 1). This effect was still clearly evident in both CpG groups after 8 weeks, with a greater effect than in the beclomethasone group (Table 1).

The comparison of the different therapy forms indicated no significant differences (Table S3).

The comparison of all horses treated with CpG with the beclomethasone therapy revealed no significant differences among the treatment forms between I and II and between I and III (Table 2).

### 3.11 Viscosity of secretion

Both CpG groups showed a significant improvement in the viscosity of the tracheal mucus directly after treatment and sustained over 8 weeks (Table 1; Figure 1). The beclomethasone group showed no improvement (Table 1; Figure 1).

The comparison of the different therapy forms indicated no significant difference (Table S3).

The comparison of all horses treated with CpG with the beclomethasone group revealed a significant difference on the part of the CpG treatment between I and II and between I and III (Table 2).

### 3.12 Neutrophilic granulocytes in the BAL

All three treatment forms resulted in a significant reduction in the neutrophilic granulocytes in the BAL directly after treatment (Table 1; Figure 1). Furthermore, the CpG groups also showed a significant improvement after 8 weeks in contrast to the beclomethasone group (Table 1; Figure 1).

The comparison of the three therapy forms showed no significant differences (Table S3).

The comparison of all horses treated with CpG and the beclomethasone group revealed a significant difference on the part of the CpG therapy after 8 weeks (Table 2).

### 3.13 HOARSI

Both CpG groups attained a significant improvement in more than half of the HOARSI scores after 8 weeks (Table 1; Figure 1). This was also reflected in the large effect size (Table 1).

The comparison between CpG and beclomethasone treatment revealed a significant improvement on the side of the CpG therapy in its long‐term effect (Table S3). There was no significant difference determined between the two CpG groups.

The comparison of all horses treated with CpG and the beclomethasone group showed a significant difference in favour of the CpG treatment (Table 2).

### 3.14 Laboratory diagnostic parameters

There were no significant differences among the therapy groups in any of the cytokines examined (IL‐4, IL‐10, IL‐17, and interferon‐gamma [IFN‐γ]) (Table S3). The comparison between all horses treated with CpG with the beclomethasone group demonstrated no significant difference neither between I and II, nor between I and III for any of the cytokines examined.

**Figure 2:**
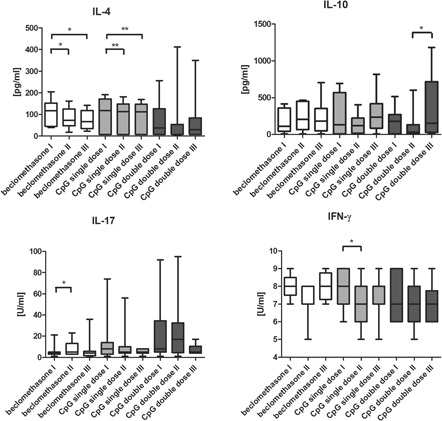
IL‐4 (pg/mL), IL‐10 (pg/mL), IL‐17 (U/mL), and IFN‐γ (U/mL) concentration in BAL samples of equine asthma‐affected horses at first examination before inhalation treatment (I), after 10 inhalations (II) of beclomethasonee (n = 9 equine asthma‐affected horses), CpG single dose (CpGsd) (n = 11 equine asthma‐affected horses) or CpG double dose (CpGdd) (n = 9 equine asthma‐affected horses) and after 8 weeks without any treatment to evaluate long‐term effect (III). Calculated value of *P* < 0.05 displayed as *, *P* < 0.01 as **. BAL, bronchoalveolar lavage; IFN‐γ, interferon‐gamma; IL, interleukin

IL‐4 decreased significantly in the CpGsd and beclomethasone group directly after treatment and after 8 weeks (Table 1; Figure 2). The beclomethasone group showed the greatest effect here (Table 1).

IL‐10 decreased in both CpG groups directly after treatment (Table 1; Figure 2). After 8 weeks, the CpGdd group showed a 100% increase (Table 1). The beclomethasone group showed an increase of 25% directly after treatment and after 8 weeks (Table 1).

IL‐17 increased by 50% in the beclomethasone group directly after treatment (Table 1). The CpGsd showed a decrease, and the CpGdd showed nearly no change (Table 1; Figure 2). After 8 weeks, both CpG groups displayed a decrease by approximately 70%, while the beclomethasone group showed a slight increase in comparison to the original value (Table 1).

IFN‐γ showed a minimal decrease in all three groups directly after treatment (Table 1). This was also evident in both CpG groups after 8 weeks (Table 1). The beclomethasone group returned to the original value after 8 weeks (Table 1: Figure 2).

### 3.15 Results of the mRNA analysis from CD4^+^ lymphocytes

**Figure 3:**
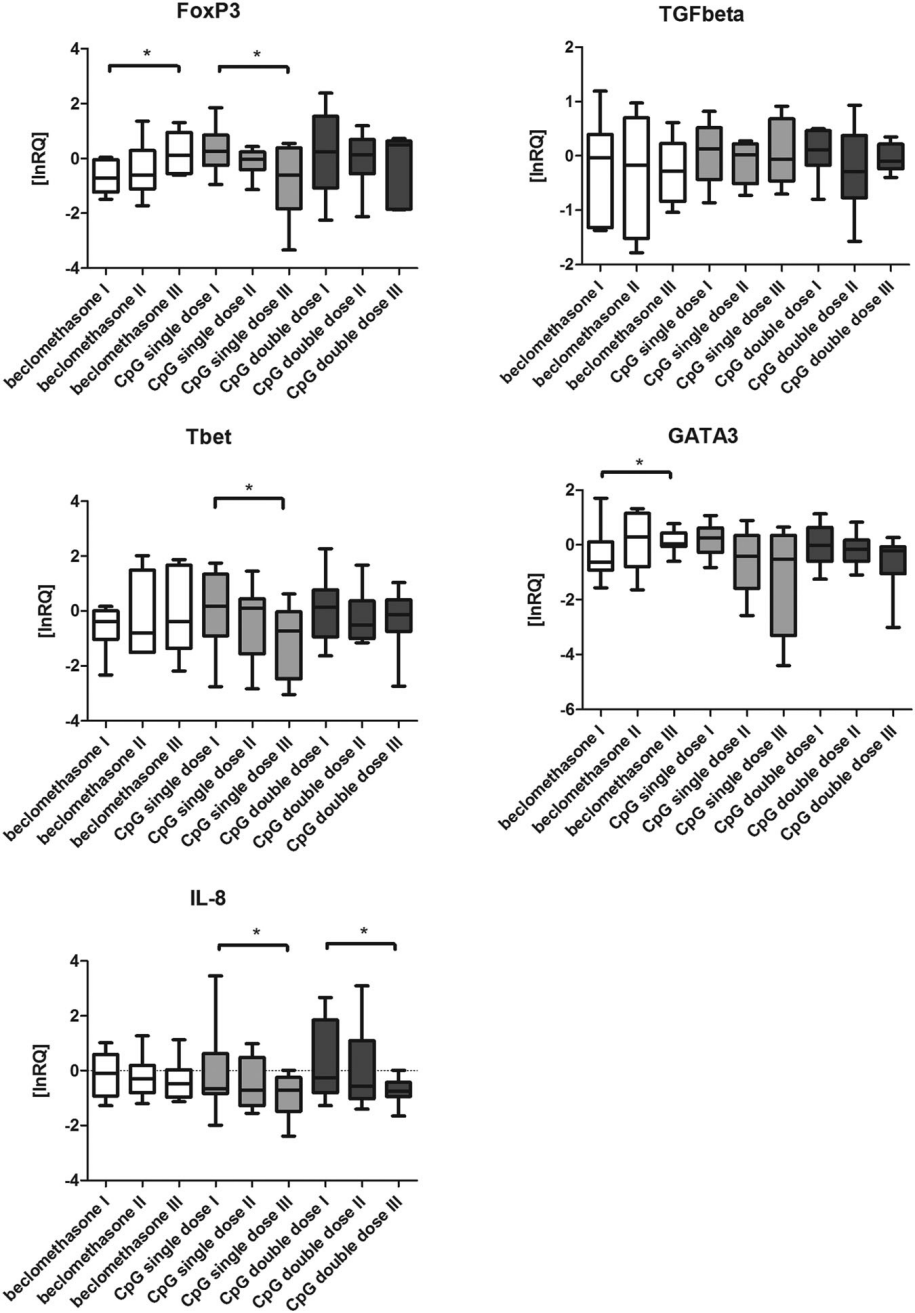
FoxP3, TGF‐β, T‐bet, GATA3, IL‐8 m‐RNA concentration via real‐time PCR from CD4+ cells in BAL samples of equine asthma‐affected horses. The values refer to a reference gene (BGus), which was used for calibration. For calculation, the logarithm of the relative quantification (lnRQ values) was used. First examination before inhalation treatment (I), second examination (II) after ten inhalations of beclomethasone (n = 9 equine asthma‐affected horses), CpG single dose (CpGsd) (n = 11 equine asthma‐affected horses) or CpG double dose (CpGdd) (n = 9 equine asthma‐affected horses) and third examination (III) after eight weeks without any treatment to evaluate long‐term effect. Calculated value of *p *<  0.05 displayed as *

The results of the mRNA analysis (FoxP3, TGF‐β, T‐bet, GATA‐3; IL‐8) from the CD4^+^ lymphocytes of the BAL are portrayed in Figure 3 and Table 1. The comparison of treatments indicated no significant differences among the treatment forms, except in T‐bet between I and II between CpGsd and CpGdd (Table S3). There were also no significant differences between all horses treated with CpG and the beclomethasone group in the parameters examined (Table 2).

FoxP3 decreased significantly after 8 weeks in CpGsd group (Table 1; Figure 3). The beclomethasone group showed a significant increase after 8 weeks (Table 1; Figure 3). The CpGsd showed a significant decrease of FoxP3 after 8 weeks (Table 1; Figure 3).

TGF‐β decreased minimally in all three groups directly after treatment (Table 1; Figure 3). After 8 weeks, the values of all three groups were close to the original values (Table 1; Figure 3).

The beclomethasone group showed a minimal increase at the second and third examinations in T‐bet expression (Table 1; Figure 3). Both CpG groups showed a decrease in T‐bet after 8 weeks, which was significant in CpGsd (Table 1; Figure 3).

GATA‐3 decreased minimally but not significant in both CpG groups directly after treatment and after 8 weeks (Table 1; Figure 3). The beclomethasone group showed an increase, which was significant after 8 weeks (Table 1; Figure 3).

IL‐8 decreased minimally in both CpG groups directly after treatment (Table 1; Figure 3). After 8 weeks, both CpG groups showed a significant decrease (Figure 3).

## 4 DISCUSSION

The current study aimed to compare two different dosages of the CpG‐GNP inhalation therapy, to examine its efficacy and to evaluate a possible long‐term effect over 8 weeks. The results were compared to a once daily corticosteroid inhalation treatment. The hypothesis was that the immunmodulatory CpG treatment has an ongoing long‐term effect over 8 weeks without further medication or management strategies in a natural environment. This was fulfilled. The dose‐response comparison of the CpGsd (single dose) and the CpGdd (double dose) enabled the next step in the direction of dose‐finding (Phase IIb), after reviewing the therapy concept (Phase IIa) of a previous study.^16^ The double CpG dose showed no significant advantages over the single CpG dose in the majority of the parameters examined. Therefore, doubling the dosage seems neither necessary nor advisable. The loss of effect for some parameters when doubling the dose of CpG could be a result of local saturation level. In mice higher dosages per kg body weight were used via i.v. injection (50 µg per mouse)^19^ which indicate in our understanding that this is not a detrimental effect of the drug or the vehicle at high dose. Furthermore, the study aimed to evaluate the immunological effects of the Th‐1 and Th‐2 immune response, as well as the regulatory T cells. In human medicine, CpG administration has been shown to reduce Th2 immune response and activate T regulatory cells.^35^ On the immunological level, a significant decrease of the Th2 cytokine IL‐4 and the Th1 cytokine IFN‐γ was indicated. The mRNA analysis showed that the CpG therapy resulted in a decrease of IL‐8, T‐bet, and GATA‐3‐mRNA expression.

Most preparations of corticosteroid inhalations should ideally be administered every 12 hours, which is often not possible for many horse owners.^36^, ^37^ For economic reasons, this was also not possible within the scope of this study, since the horses were located in their home stables and the inhalations were conducted by the veterinarians (SG, CB), to ensure that the treatments were conducted properly. In addition, Niedermaier and Gehlen described a clinical sufficient effect of once a day inhalation treatment of a corticosteroid in many patients.^36^ However, for a better comparability it would be recommendable to treat all groups with the same interval. This is the aim for the next study. The CpG treatment was performed in 48 hour intervals according to the former protocol.^16^ The number of inhalations was the same for both treatment regimens, and the follow‐up examination was conducted directly following the final treatment for both groups, to enable an accurate comparison of the treatments.

Inhalative administration of the medication was chosen, to reduce potential systemic side effects and use the advantage of the topical, inhalative administration with its locally restricted effect and targeted application at the site of pathogenesis.^36^, ^38^ The target cells are those with TLR9 receptors, such as pulmonary neutrophils, bronchial epithelial cells, capillary endothelial cells and macrophages.^18^

GNPs have proven to be a safe and effective carrier system for the CpG inhalations.^15^, ^16^, ^19^, ^20^, ^22^ Through variations in the production of the GNP, the previous production process could be optimized and designed more efficiently.^21^ Based on the results of Zillies et al,^39^ Geh et al also developed a storage stable and sterilised lyophilisate of the CpG‐GNP dispersion, which in future will make its storage and application much easier and more practicable.^40^

To reduce the effects of seasonal fluctuations on the therapy effect as much as possible, the patients of all three treatment regimens were treated in parallel. In this way, the influence of external conditions on the effect of the different treatment forms was minimised.

Due to the fact that there are no data supporting a long‐lasting effect of inhaled corticosteroids in asthmatic horses,^41^ this means that the observed improvement 8 weeks after the end of the treatment with beclomethasone could also be due to other factors (eg, season, weather, hay quality), that are likely present in the CpG groups and during the treatment period. No side effects were evident in any of the horses of the three groups, which would have required the discontinuation of the study. The fibrinogen values measured also showed no indication of an inflammatory response resulting from the treatment (data not shown). These results reinforce the finding gained by the previous studies, that the CpG‐GNP is well tolerated by the patients.^15^, ^16^, ^20^

The CpG therapy provides a new alternative for horses, which should not be treated with corticosteroids^42^ due to pre‐existing conditions (eg, PPID, equine metabolic syndrome, laminitis).

The CpG inhalations, in contrast to the corticosteroid therapy, succeeded in significantly sinking the respiratory rate at rest, as well as regarding the long‐term effect.

Since breathing type is an external sign of the restriction of the airways,^42^ the improvements in the beclomethasone and CpGsd groups are of great clinical relevance.

In addition to the breathing type, nasal flaring was evaluated. Nasal flaring is the result of increased breathing activity, as well as a mechanism for the reduction of respiratory resistance.^43^ All therapy forms attained significant improvements, as well as regarding the long‐term effect. Therefore, in connection with further pulmonary function tests, a reduction of breathing difficulty and thus an improvement in general condition can be determined in the horses treated in all three groups.^43^

The reduction of nasal discharge in the treatments agrees with the findings of the endoscopic examination. The occurrence of mucus nasal discharge, especially when accompanied by occasional coughing, can be an early clinical indication for a higher risk of equine asthma,^44^, ^45^ and can be used as a good indicator for the development of the disease by the owner or in the clinical examination.^46^

The partial pressure of oxygen was determined to evaluate the oxygen supply. In all three forms of treatment, a significant increase was identified in the partial pressure of oxygen (PaO_2_) measurements, and also after the evaluation of the long‐term effect. The increase in PaO_2_ values in connection with reduced levels of interpleural pressure could be a clear indication of an improvement of the pulmonary ventilation.^6^

The PaO_2_ can be subject to certain variations, depending on geographical location (elevation over NHN) and weather influences (high or low pressure), since it is dependent on the predominant barometric pressure.^26^ For this reason, in addition to the PaO_2_ in connection with the predominant barometric pressure at the time of the examination, the alveolar arterial oxygen gradient was also calculated.^26^ The arterial partial oxygen pressure, as well as the alveolar arterial gradient, revealed greater clinically relevant results from the CpG inhalations than from the established beclomethasone therapy. The larger improvement is in part due to the higher baseline values of PaO_2_ for the beclomethasone. Since both parameters (PaO_2_ and AaDO_2_ gradient) reach a plateau in health, there was less improvement possible in this group, regardless of the medication efficacy. In a previous study, no statistically significant long‐term effect was identified after five inhalations with the single CpG dose.^16^ However, the current study showed that increasing the number of inhalations with CpG‐GNPs to 10 resulted in a positive effect on both the PaO_2,_ as well as the AaDO_2_, which was still evident after 8 weeks.

In a previous study, no statistically significant long‐term effect was identified after five inhalations with the single CpG dose.^16^ However, the current study showed that increasing the number of inhalations with CpG‐GNPs to 10 resulted in a positive effect on both the PaO_2,_ as well as the AaDO_2_, which was still evident after eight weeks.

The measurement of the interpleural pressure gives an indication of the degree of severity of the current bronchoconstriction.^47^ To compensate for the constriction of the airways and prevent hypercapnia, the interpleural pressure increases. Therefore, an abdominally‐forced breathing effort becomes necessary during bronchoconstriction, in which the expiratory pressure is significantly increased, and the interpleural pressure increases as a result.^6^ All three treatment regimens attained a short‐ and long‐term reduction of the indirectly measured pulmonary pressure. Improving the interpleural pressure should reduce the airway resistance with a reduction of the bronchospasm.^6^

In the entirety of the three treatment groups, the final mean value for the interpleural pressure was still somewhat above the physiological range of <10 cmH_2_O. This could be associated with the airway remodelling in the lungs, which occurs especially due to neutrophilic inflammatory reactions, epithelial proliferation, and smooth muscle proliferation.^4^, ^6^ These reconstruction processes make a full regeneration of the tissue very unlikely in a longer‐existing illness.^43^ Since 79% of the horses in this study had already suffered from the disease for 5 years or longer, one must assume at least partially irreversible lung damage.

Excessive accumulation of mucus in the airways is an important indicator of equine asthma,^25^, ^48^ which can be caused by increased secretion or by reduced mucociliary clearance.^25^

All three forms of treatment showed a long and short‐term reduction of the quantities of mucus, evaluated endoscopically. In agreement with the results of previous studies,^16^ the treatment with CpG reduced the quantity of mucus, resulting in an improvement of the symptoms such as mucus‐induced coughing or breathing difficulty, as these are directly correlated.^6^, ^49^

In horses that suffer from equine asthma, there is a high correlation between the accumulation of mucus and the percentage of neutrophils in the BAL.^50^ Neutrophilic inflammation of the airways can cause the increased production of mucus, due to the increased secretion of mucines, among other things.^50^, ^51^ Because a pathological increase in the quantity of secretion is also caused by oxidative processes of neutrophilic inflammation,^52^ a decrease in the quantity of mucus can also be considered an anti‐inflammatory effect of the CpG treatment.

The reduction of the quantity of the mucus also has a positive influence on the improvement of the partial oxygen pressure, since a smaller quantity of mucus in the airways also improves the gas exchange in the lungs.

Besides the quantity of mucus, its viscosity was also evaluated. In contrast to the therapy with beclomethasone, a significant reduction of the viscosity of the mucus was evident after the inhalations with both CpG concentrations. In clinical exacerbation of equine asthma, the mucus becomes more viscous,^25^ so that an improvement of the viscosity indicates that the therapy could improve mucociliary clearance. Furthermore, the reduction of mucous secretion can be expected due to the reduced quantity of mucus from the CpG treatment.^25^ Herholz et al^53^ could also determine a positive correlation between the reduction of the mucus viscosity and the results of the pulmonary function tests.

The percentage of neutrophilic granulocytes in the total cell count of the BAL is evaluated, as the most important indicator of an inflammation of the lower airways.^3^, ^6^ The results indicate that only the CpG therapy can achieve a significant sustained anti‐inflammatory effect over 8 weeks. The reduction of pulmonary inflammation is one of the most important cornerstones in the treatment of equine asthma.^3^ An increased accumulation of neutrophils in the airways due, among other things, to oxidative stress, creates an environment producing increased mucus production and bronchospasm.^6^, ^11^ It is already known that treatment with corticosteroids only decreases the percentage of neutrophils with concurrent environmental changes and thus can achieve an improvement in the clinical symptoms.^11^, ^54^ Therefore, the effect attained by the CpG treatments is of great importance, since the long‐term effect was actually significantly superior to that of the beclomethasone therapy, which was previously considered the most effective form of medicamentous therapy to manage clinical symptoms of equine asthma.^6^, ^55^ The therapy also achieved a long‐term improvement in the inflammatory status in the lungs. Accordingly, the percentage of neutrophils continued to drop off in the 8 weeks following the last treatment with CpG.

The owners were questioned regarding pathology and performance before the first treatment and 8 weeks after the last treatment, via the standardized HOARSI questionnaire (Horse Owner Assessed Respiratory Signs Index).^56^ The beclomethasone therapy resulted in a minimal reduction of the mean value of the scores. After the CpG treatments, however, the owners rated the condition of their horses as significantly improved. The HOARSI questionnaire is a reliable and informative assessment of the medical condition of the horse and the results of the examinations of the respiratory tract.^46^, ^57^

Since a predominant Th‐2 immune response, among others, is responsible for horses with equine asthma reacting to certain allergens with an exacerbation of the airway pathology,^58^ a goal of this study was to show that the therapy can achieve a reduction of the Th2 cytokine IL‐4. This was confirmed by the results of the beclomethasone and CpGsd group which could achieve a significant and sustained reduction of the IL‐4 concentration in the BAL. This agrees with a previous study^27^ and the results of a study from Giguere et al^54^ in which an inhalative fluticasone therapy could also effect a reduction of the allergy‐mediated Th2 cytokine. It confirms the capacity of the CpG treatment to downregulate IL‐4.

IL‐4 is also involved in the recruitment of neutrophils,^6^, ^59^ which was also decreased in the BAL by the treatments.

Tregs are attributed a critical protective role against allergies.^60^ These cells produce the anti‐inflammatory cytokine IL‐10, which has an important suppressive influence on the allergy‐mediating Th2 cytokines like IL‐4 or IL‐5, as well as an inhibitory effect on the proinflammatory Th1 cytokine IFN‐γ.^17^, ^61^, ^62^ Neither the beclomethasone nor the CpG treatments could effect a significant increase in IL‐10. A slight increase in the CpG groups, after 8 weeks could possibly indicate a delayed occurrence of immunomodulation.^62^

In other studies, an upregulation of the IL‐10 production could be achieved through stimulation with CpGs in both humans^63^ and horses.^15^, ^20^ The majority of participants in this study were horses of advanced age (86% were 15 years old or more). Robbin et al^60^ described how fewer FoxP3 positive CD4^+^ cells could be found in horses of older age.

The Th17 cytokine IL‐17 is attributed a role in the pathogenesis of equine asthma, due to its chemotactic and activating effect on neutrophils.^64^, ^65^ It is assumed that corticosteroids have an inhibitory effect on Th2 cytokines (IL‐4 and IL‐5) and IL‐8,^66^ and therefore work via other mechanisms than the Th17‐mediated path. This could also explain why the IL‐17 concentration after treatment increased significantly in the beclomethasone group in comparison to the CpG groups.

Since an increased expression of IL‐17 in the BAL of horses with equine asthma after exposure to allergens was determined in a study from Debrue et al,^64^ the nonsignificant decrease in the IL‐17 concentrations in both CpG groups regarding the long‐term effect can be considered a positive treatment response. This is contrasting to the beclomethasone therapy.

A decrease in the IL‐17 concentration, as was observed in both CpG groups, could also be associated with the simultaneously observed reduction of the neutrophilic granulocytes in the BAL through the CpG therapy and therefore a reduction of the inflammation in the lower airways.^65^

IFN‐γ is an important representative of the Th1 cytokine.^67^ IFN‐γ decreased in all three groups direct after treatment. This was significant only in the CpGsd group. These results contradict the results determined by Bohle,^67^ who described an increased release of the Th1 cytokine IFN‐γ, which should have an inhibitory effect on the Th2 cytokines IL‐4, IL‐5, and IL‐13.^68^ After 8 weeks only the CpG groups showed a nonsignificant decrease. This can, however, be considered a positive result regarding the sustained reduction of the inflammatory response and neutrophilic granulocytes in the lungs.

Studies of horses with equine asthma,^54^ as well as human asthma sufferers,^69^ determined increased IFN‐γ expression in phases of exacerbation. In a study by Horohov, IFN‐γ mRNA was also increased in equine asthma exacerbation after antigenic challenge.^30^ Since IFN‐γ can contribute to an increase in the chronic pulmonary inflammation in asthmatic illnesses,^69^ and, in combination with IL‐10 and Tregs, can also have an inhibitory effect on the Th2 immune response,^68^ the results attained for IFN‐γ must be considered in relationship to the Tregs and the clinic.^70^

The measurement of the mRNA in CD4^+^ cells serves to examine the influence of the forms of treatment on the activity of Tregs. In a study from El Abbas et al horses with equine asthma, in contrast to healthy control horses, did not react to antigenic challenge with a significant upregulation of the FoxP3 expression in the BAL.^32^ This can possibly be ascribed to the fact that the reactive capacity of Tregs in equine asthma is generally diminished.^32^ In the comparison of treatments, there were no statistically significant differences in the FoxP3 expression, although the beclomethasone group showed an increase after 8 weeks and the CpGsd showed a decrease.

Certain reasons indicate that FoxP3 is not the ideal marker to analyze the activity of Tregs. Since the transcription factor for humans is described as a marker for nonactivated regulatory T cells,^71^ it is possible that FoxP3 in horses is also more likely a measured value for nonactivated, rather than activated Tregs.

In an in vitro study of horses with insect bite hypersensitivity (IBH), the FoxP3 expression in those horses was significantly lower after antigen stimulation than in healthy horses.^72^ Since IBH, like equine asthma, is a disease with an allergic genesis, this suggests that horses with equine asthma also have a diminished ability to produce Tregs. This would explain the absence of an increase in the CpG groups. However, the beclomethasone group did show an increase after 8 weeks.

A study by Robbin et al determined that even healthy horses over 15 years old display a significantly lower percentage of FoxP3 positive CD4^+^ cells than younger horses.^60^ Therefore, one may assume that the immunological reaction of the Tregs in the horses examined was additionally reduced due to their ages. Older humans suffering from asthma also display a lower FoxP3 mRNA expression.^73^

Since the determination of Tregs ideally requires the evaluation of a combination of FoxP3 and the marker CD25 from CD4^+^ T cells, and not FoxP3 alone, a FACS analysis is better suited to make a statement on the activity of Tregs than the mRNA determination.

The regulatory function of Tregs is mediated by the cytokine TGF‐β, in addition to IL‐10 and others.^62^ The potential to influence numerous immunological reactions is attributed to this cytokine, including a direct anti‐inflammatory effect on Th1 cells as well as an influence on healing processes and fibrosis of tissue.^74^

The TGF‐β‐mRNA expression of CD4^+^ cells was measured via real‐time PCR. Here, there was no significant change of the TGF‐β‐mRNA expression at II or III for any of the three treatment groups. A study from Desjardins et al^74^ was also unable to determine differences in the TGF‐β concentration after allergenic challenge in the BAL of healthy horses or horses with equine asthma. These results indicate that TGF‐β does not play a great role in the pathophysiology of equine asthma.^74^ In human asthma, a connection could be made between an increased expression of TGF‐β and the reconstruction of the lungs in a bronchial fibrosis,^75^ although this could not yet be confirmed in horses.^74^

The transcription factor T‐bet could have great significance for the mediation of the effect of the CpG, since its activation promotes Th1‐associated cytokines and simultaneously inhibits Th2 associated cytokines.^76^ T‐bet is considered in human medicine to be a “fate‐deciding” factor for a Th1 immune response,^77^ and in this is significantly influenced by the cytokines IL‐4 and IFN‐γ. It can be considered the counterpart to the transcription factor GATA‐3, since this factor is influenced by the same cytokines and can induce a Th2‐dominated immune response when activated.^77^ In addition, it influences both the acquired immune system, as well as the innate immune system.^78^ The CpGsd showed a reduction of the T‐bet expression after 8 weeks. In connection with the decrease in IFN‐γ, IL‐4 and GATA‐3, this is also considered a positive balancing immunomodulatory effect in the CpGsd group, in which neither Th1 nor Th2 dominates. One must consider the results for T‐bet in this relationship in the context of the results for the Th2 cytokine IL‐4. Here, both the beclomethasone group and the CpGsd group could achieve a significant reduction of the IL‐4 concentration directly after treatment, analogous to the results of a study by Giguere, in which an inhalative fluticasone therapy also effected a decrease in the allergy‐mediating Th2 cytokine.^54^

The slight change in the expression of the transcription factor T‐bet, with the exception of the CpGsd group, could also be the result of a deficit of T‐bet in horses suffering from equine asthma over a long period of time, as described by Vale‐Pereira et al^73^ in human long‐term sufferers of asthma.

In human asthma, a significant role of the transcription factor GATA‐3 was determined for a Th2‐dominated phenotype.^79^ Therefore, GATA‐3 has already been evaluated as a target protein in asthma therapy for humans.^79^ An increase from GATA‐3, as in the beclomethasone group after 8 weeks, would therefore be considered unfavourable for the progression of the disease. In contrast, the CpG group showed a decrease. However, a study by Couetil et al^80^ could not determine any difference in the GATA‐3 activity between healthy horses and horses with equine asthma.

The minor change in the GATA‐3 expression could be associated with the ages of the study's subjects and their resulting immunosenescence, or the age‐related decreased efficiency of the immune systems, which has been especially detected in the lungs.^73^, ^81^

The mRNA expression of the IL‐8 cytokine, which serves as an inflammatory mediator and plays a particular role in the chemotactic recruitment of neutrophils,^54^ was significantly reduced in both CpG groups after 8 weeks. A part of the effect of corticosteroids is generally attributed to their inhibitive influence on the expression of IL‐8, amongst others.^66^ This observation was not made in this study.

In a study by Giguere et al^54^ a positive correlation between the IL‐8 concentration and the percentage of neutrophils could be established. Similar results could be achieved in both CpG groups, which showed a significant decrease in IL‐8 after 8 weeks. The neutrophilic granulocytes also sank significantly after treatment, and this effect was also sustained after 8 weeks. Thus, the therapy achieved an anti‐inflammatory effect, which is one of the most important goals in the therapy for equine asthma.

The summarizing analysis of all clinical results directly after the last inhalation determined that the CpGsd resulted in a significant improvement in 82% of the parameters, while the CpGdd therapy had a significant, positive effect on 72% of the parameters. The corticosteroid treatment with beclomethasone also showed a significant improvement in 72% of the clinical parameters.

In the long‐term evaluation, the CpGsd showed a significant, sustained improvement in 100% of the parameters in comparison to the initial values, while the CpGdd showed a significant improvement in 67%, and the corticosteroid therapy in only 50% of the parameters.

In comparison to the preceding studies considering the long‐term effect, the current study reveals that 10 inhalations resulted in medium to high clinical effects on 100% of the parameters upon 8 weeks after the final treatment (Table S5). Seven inhalations^22^ showed middle to high clinical effects on 80% of the measured values upon 6 weeks after treatment. In the study, in which the patients received five CpG inhalations,^16^middle to high clinical effects were measured in 50% of the parameters 4 weeks after the final treatment. These results indicate that more frequent inhalations work effectively over a longer period of time (8 weeks).

Through the reduction of their symptoms with the CpG therapy, the horses return to a higher performance potential, and can even be trained more intensively and used for riding, which was often not possible before the therapy, due to the associated limitations of the disease. Particularly striking is the indication that the inhalation with CpG‐GNP, especially considering the sustained therapeutic effect, has an advantage over the corticosteroid therapy. With a longer sustained effect, the intervals between treatments could be extended.

The interpretation of the cytokine results is difficult, since the individual values can vary greatly among different horses.^30^, ^81^ An evaluation of repeated boosters could reveal more, for example after 3 to 4 weeks or as needed dependent on symptoms. Repeated stimulation of the immune system with CpG could enhance the immunomodulatory effect through the induction of IL‐10.^67^ This could lead to the creation of memory cells, which could result in a stronger immune response upon renewed contact.^66^

This therapy concept could also be relevant for other allergic diseases, such as IBH and multiple hypersensitivities.^82^, ^83^

Equine asthma is a naturally occurring animal model for human allergic neutrophilic asthma.^6^ Therefore, the application and evaluation of this immunomodulatory inhalative treatment is of great interest for human allergic asthma, and could establish a new treatment option beyond solely symptomatic treatment.

## CONFLICT OF INTERESTS

The authors declare no competing financial interests. The study was conducted at the Equine Clinic, Centre for Clinical Veterinary Medicine, LMU Munich, Germany. Part of the lab work was performed by CB and JK in the lab of the Institute for Animal Welfare, Ethology, Animal Hygiene and Animal Husbandry, Faculty of Veterinary Medicine, Department of Veterinary Science, Ludwig‐Maximilians‐University, Munich. An abstract of this study was presented in part at the 2016 British Equine Veterinary Association (BEVA) Congress, Birmingham, England.

## Supporting information

Supporting information
