# Supplementary material for: Immunomodulatory asthma therapy in the equine animal model: A dose‐response study and evaluation of a long‐term effect
Source: Immun Inflamm Dis. 2019 May 29;7(3):130–49. doi: 10.1002/iid3.252 (PMC6688086; doi:10.1002/iid3.252)
Supplement: Supplementary file 1 — Supporting information [file IID3-7-130-s001.docx]

## Supplements

**Clinical and laboratory evaluations**

The variables examined included the measurement of the following: respiratory rate at rest; breathing type (relative units [RU] in the range of 0 to 4, where 0 = no or little movement in the ventral region of the flank and 4 = obvious abdominal lift, heave line and nasal flaring) (Gerber et al. 2000); nasal discharge (RU in the range of 0 to 3, where 0 = none, 1 = mild, 2 = moderate and 3 = severe discharge); auscultation of lungs and trachea (in the range of RU 0 to 4, where 0 = physiological, 1 = mild, 2 = moderate, 3 = severe and 4 = wheezes/rhonchi) (Robinson 2001, Klier et al. 2012, 2015); and arterial blood gas parameters (Grabner 2005). Nasal flaring, the clinical sign of equine asthma, was also categorised in the range of 1 to 4, where 1 = physiological movement of the nostrils, and 4 = extreme widening of the nostrils for each breath (Robinson, Olszewski et al. 2000).

The partial pressures of oxygen and carbon dioxide (PaO2. PaCO2) were determined. The physiological values were set at 100 mmHg ± 5 mmHg for PaO2 and 40 mmHg ± 5 mmHg for PaCO2 (Grabner 2005). The arterial blood gas values were measured via the IRMA® blood analysis system.^d^ The alveolar-arterial oxygen gradient (AaDO2) was calculated according to the current atmospheric pressure and the measured blood gas values [AaDO2= (atmospheric pressure -47 mmHg) x 0.2095- PaCO2 - PaO2] (Grabner 2005). In addition, an endoscopic examination and quantification of secretion (grade 0–5. 0 = none, clean singular tracheobronchial secretion [TBS] to 5 = extreme, profuse amounts) (Gerber et al. 2004) and viscosity (grade 1 = fluid to 5 = viscous) (Gerber et al. 2004) of the TBS was performed. Cytological examinations of the bronchoalveolar lavage (BAL) samples were performed to determine the percentage of neutrophils in the total cell count after staining with Diff-Quick solution.^e^ Indirect measurement of the maximum interpleural pressure differences (⧍Pplmax) was performed by a Venti-Graph^f^ via esophageal probe (Deegen and Klein 1987).

In order to evaluate the progression of the disease, the horse owner assessed respiratory signs index (HOARSI) was used (Gerber et al. 2011). This was evaluated via a standardised questionnaire completed by the horse owners at the initial examination (I) and final examination (III). The criteria evaluated were coughing, nasal discharge, respiratory rate at rest and during work, regulation of respiration, and performance and willingness of the horses to work. The horses were categorised in the HOARSI groups 1 to 4 (Gerber et al. 2011). The patients in HOARSI 1 were not included in the study, since these horses did not show the clear presence of a chronic lung disease, or were possibly in remission. HOARSI 2 showed minimal respiratory symptoms of the disease, HOARSI 3 moderate symptoms, and HOARSI 4 severe respiratory symptoms (Gerber et al. 2011).

In the run-up to the study, compatibility criteria were specified, which would lead to the termination of treatment should they appear. These are delineated in the protocol of the “Veterinary co-operative oncology group - common terminology criteria for adverse events” (VCOG-CTCAE) (Klier 2011, VCOG 2011, Wärther 2013).

^d^ Diametrics Medical incorporated, Keller Medical, Bad Soden, Germany

^e^ Medion diagnostics, Düdingen, Switzerland

^f^ Boehringer Ingelheim, Germany

**Magnetic cell sorting (MACS)**

One half of the BAL sample was filtered through a 100µm cell strainer (Miltenyi Biotec GmbH). This eliminated cells with larger diameters (>100µm). Based on the varying sizes of different cell types, the filtration ensured that the sample consisted primarily of lymphocytes (Kleiber et al. 2005). Lymphocytes have an average size of ca. 10µm (Grabner 2005).

The filtered cell suspension was washed with 10ml MACS buffer (MACS BSA stock solution and autoMACS rinsing solution; Miltenyi Biotech, Bergisch Gladbach), then centrifuged for 10 minutes at 300g, and finally the supernatant removed and discarded. The remaining cell pellet was incubated in the refrigerator for 20 minutes with 10µl of an equine CD4 antibody (Mouse anti Horse CD4: RPE, Monoclonal Antibody, Clone: CVS4, Isotype: IgG1, Firma Bio-Rad AbD Serotec GmbH, Puchheim). Then 1 ml MACS buffer was added, mixed with a pipette and centrifuged for 10 minutes at 300g, after which the supernatant was discarded. 80µl MACS buffer was added to the cell pellet, mixed with the aid of a pipette and finally brought into suspension with 20µl MACS antibodies (Anti-Mouse IgG1 MicroBeads, Miltenyi Biotech GmbH, Bergish Gladbach). The sample was incubated in the refrigerator (4°C) for 15 minutes. It was then washed again with 1 ml of the MACS buffer and centrifuged for 10 minutes at 300g. The supernatant was again discarded, and the cell pellet resuspended with 500µl MACS buffer. For the cell separation, the Quadro MACS™ Separator System was used together with the LS Columns from Miltenyi Biotec GmbH.

For the preparation of the columns, the LS columns were rinsed with 3ml of the MACS buffer and the liquid to be discarded was collected in 15ml Falcon tubes. The cell suspension was pipetted into the columns, and rinsed three times with 3ml MACS buffer used for each rinse. A new rinse was only started when the column was free from liquid. The LS column used was then removed from the magnet and set on a new 15ml Falcon tube. Then, 5 ml of the MACS buffer was pipetted in and pushed through the column using the syringe plunger. Because the cells that were not bound to the CD4 antibodies were removed in the previous steps, the suspension contained only the separated CD4 T lymphocytes at this stage.

For the transfer of the cells in the RNA-stabilisation medium (RNAlater; Qiagen GmbH, Hilden), the suspension was centrifuged for 10 minutes at 300g and the supernatant then discarded. The cell pellet was then suspended with 200µl PBS and finally brought into suspension with 1800µl RNAlater.

The sample was incubated in the preservative medium in the refrigerator at 4°C overnight, before it was divided into four cryotubes each containing a 500µl sample. The samples were stored at -80°C until further processing.


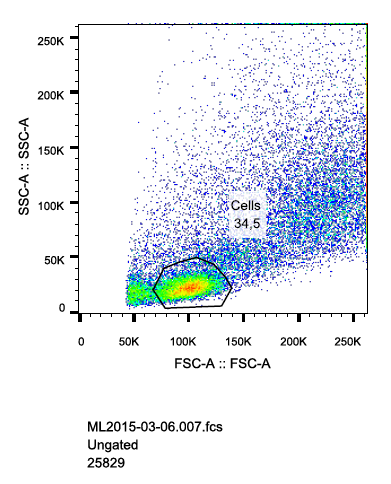


**Figure 4:** Result of the flow cytometry in consideration of all BAL cells by forward scatter (x-axis) and side scatter (y-axis) (produced in collaboration with Dr. Michael Lehmann, Institute for Infectious Disease and Zoonoses, LMU Munich)


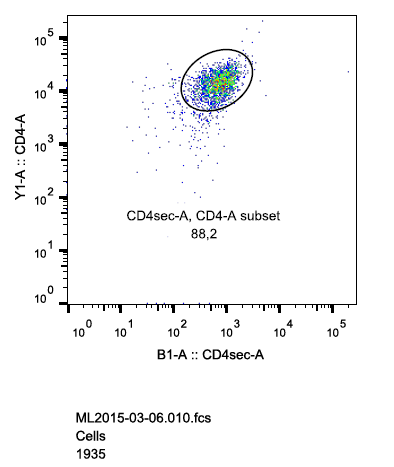


**Figure 5:** Results of the FACS analysis. Represented here are the CD4 cells stained with FITC. the cell population of interest is circled (produced in collaboration with Dr. Michael Lehmann, Institute for Infectious Disease and Zoonoses, LMU Munich)


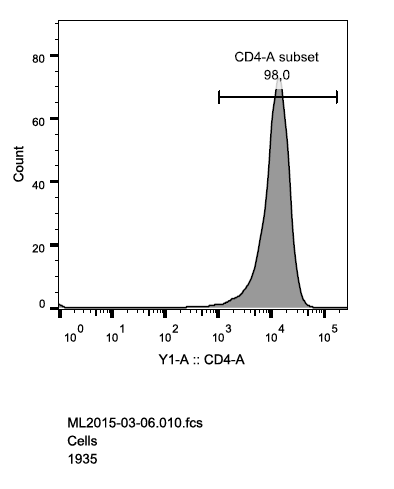


**Figure 6:** Results of the FACS analysis: 98% of the stained cells belong to the CD4+ cell population (produced in collaboration with Dr. Michael Lehmann, Institute for Infectious Disease and Zoonoses, LMU Munich)

**RNA Isolation**

The BAL cells were divided into two aliquots of 900µl each, and then centrifuged for 10 minutes at 15000g. Finally, the RNA-stabilisation medium (RNAlater) was removed. Trizol (acid guanidinium thiocyanate-phenol dissociations buffer) was added to the remaining cell pellet, in order to lyse the cells (Chomczynski and Sacchi 1987). The isolation of the RNA was completed via the standardised phenol-chlorophorm RNA extraction technique (Horohov, Beadle et al. 2005).

**Multiplex Assay ELISA**

Each cytokine has a specified individual detection limit for the measurement in the multiplex assay (IL-4: 40 pg/ml to 80.000 pg/ml; IL-10: 15 pg/ml to 35.000 pg/ml; IL-17: 10 pg/ml to 10.000 U/ml; IFN-y: 10 U/l to 5000 U/ml; IFN-a: 12 pg/ml to 30.000 pg/ml)

**Results**

**Table 3:** Comparison of the effects of the treatment forms on clinical, cytological and laboratory parameters under the determination of the p-value after Bonferroni correction

| **Examination Stage** | **Treatments in Comparison** | **P-value (Respiratory Rate at Rest)** |
| --- | --- | --- |
| I – II | Beclomethasone - CpG single dose | 0.0153 |
|  | Beclomethasone - CpG double dose | 0.1029 |
|  | CpG single - CpG double dose | 0.9273 |
| I – III | Beclomethasone – CpG single dose | 0.0237 |
|  | Beclomethasone – CpG double dose | 0.0393 |
|  | CpG single – CpG double dose | >0.9999 |

| **Examination Stage** | **Treatments in Comparison** | **P-value (Breathing Type)** |
| --- | --- | --- |
| I – II | Beclomethasone - CpG single dose | 0.9939 |
|  | Beclomethasone - CpG double dose | 0.6519 |
|  | CpG single - CpG double dose | 0.3594 |
| I – III | Beclomethasone – CpG single dose | 0.7614 |
|  | Beclomethasone – CpG double dose | 0.7689 |
|  | CpG single – CpG double dose | 0.522 |

| **Examination Stage** | **Treatments in Comparison** | **p-Value (Nasal Flaring)** |
| --- | --- | --- |
| I – II | Beclomethasone - CpG single dose | 0.1929 |
|  | Beclomethasone - CpG double dose | >0.9999 |
|  | CpG single - CpG double dose | 0.6366 |
| I – III | Beclomethasone – CpG single dose | >0.9999 |
|  | Beclomethasone – CpG double dose | >0.9999 |
|  | CpG single – CpG double dose | >0.9999 |

| **Examination Stage** | **Treatments in Comparison** | **P-value**  **(Nasal Discharge)** |
| --- | --- | --- |
| I – II | Beclomethasone - CpG single dose | >0.9999 |
|  | Beclomethasone - CpG double dose | 0.5154 |
|  | CpG single - CpG double dose | 0.8367 |
| I – III | Beclomethasone – CpG single dose | 0.2616 |
|  | Beclomethasone – CpG double dose | 0.6279 |
|  | CpG single – CpG double dose | >0.9999 |

| **Examination Stage** | **Treatments in Comparison** | **P-value (Auscultation)** |
| --- | --- | --- |
| I – II | Beclomethasone - CpG single dose | 0.4434 |
|  | Beclomethasone - CpG double dose | >0.9999 |
|  | CpG single - CpG double dose | 0.3969 |
| I – III | Beclomethasone – CpG single dose | 0.1878 |
|  | Beclomethasone – CpG double dose | 0.1158 |
|  | CpG single – CpG double dose | 0.5097 |

| **Examination Stage** | **Treatments in Comparison** | **P-value (PaO2)** |
| --- | --- | --- |
| I – II | Beclomethasone - CpG single dose | 0.6705 |
|  | Beclomethasone - CpG double dose | 0.3762 |
|  | CpG single - CpG double dose | 0.1656 |
| I – III | Beclomethasone – CpG single dose | 0.8871 |
|  | Beclomethasone – CpG double dose | 0.4923 |
|  | CpG single – CpG double dose | >0.9999 |

| **Examination Stage** | **Treatments in Comparison** | **P-value (AaDO2)** |
| --- | --- | --- |
| I – II | Beclomethasone - CpG single dose | 0.8136 |
|  | Beclomethasone - CpG double dose | 0.0366 |
|  | CpG single - CpG double dose | 0.4851 |
| I – III | Beclomethasone – CpG single dose | 0.1899 |
|  | Beclomethasone – CpG double dose | 0.2409 |
|  | CpG single – CpG double dose | >0.9999 |

|  | **Treatments in Comparison** | **P-value (Interpleural Pressure)** |
| --- | --- | --- |
| I – II | Beclomethasone - CpG single dose | 0.4833 |
|  | Beclomethasone - CpG double dose | 0.3231 |
|  | CpG single - CpG double dose | >0.9999 |
| I – III | Beclomethasone – CpG single dose | 0.5133 |
|  | Beclomethasone – CpG double dose | 0.5733 |
|  | CpG single – CpG double dose | 0.3228 |

| **Examination Stage** | **Treatments in Comparison** | **P-value (Quantity of Tracheal Secretion)** |
| --- | --- | --- |
| I – II | Beclomethasone - CpG single dose | >0.9999 |
|  | Beclomethasone - CpG double dose | >0.9999 |
|  | CpG single - CpG double dose | >0.9999 |
| I – III | Beclomethasone – CpG single dose | 0.6291 |
|  | Beclomethasone – CpG double dose | 0.2472 |
|  | CpG single – CpG double dose | 0.8733 |

| **Examination Stage** | **Treatments in Comparison** | **P-value (Viscosity of Mucus)** |
| --- | --- | --- |
| I – II | Beclomethasone - CpG single dose | 0.0711 |
|  | Beclomethasone - CpG double dose | 0.1602 |
|  | CpG single - CpG double dose | 0.4296 |
| I – III | Beclomethasone – CpG single dose | 0.1212 |
|  | Beclomethasone – CpG double dose | 0.1215 |
|  | CpG single – CpG double dose | >0.9999 |

| **Examination Stage** | **Treatments in Comparison** | **P-value (Neutrophils) BAL** |
| --- | --- | --- |
| I – II | Beclomethasone - CpG single dose | 0.8922 |
|  | Beclomethasone - CpG double dose | >0.9999 |
|  | CpG single - CpG double dose | 0.9786 |
| I – III | Beclomethasone – CpG single dose | 0.9786 |
|  | Beclomethasone – CpG double dose | 0.1551 |
|  | CpG single – CpG double dose | >0.9999 |

| **Examination Stage** | **Treatments in Comparison** | **P-value (HOARSI)** |
| --- | --- | --- |
| I–III | Beclomethasone - CpG single dose | 0.0411 |
|  | Beclomethasone - CpG double dose | 0.0813 |
|  | CpG single - CpG double dose | >0.9999 |

| **Examination Stage** | **Treatments in Comparison** | **P-value (IL-4) BAL** |
| --- | --- | --- |
| I – II | Beclomethasone - CpG single dose | 0.4302 |
|  | Beclomethasone - CpG double dose | >0.9999 |
|  | CpG single - CpG double dose | >0.9999 |
| I – III | Beclomethasone – CpG single dose | 0.0642 |
|  | Beclomethasone – CpG double dose | 0.4032 |
|  | CpG single – CpG double dose | 0.231 |

| **Examination Stage** | **Treatments in Comparison** | **P-value (IL-10) BAL** |
| --- | --- | --- |
| I – II | Beclomethasone - CpG single dose | 0.2949 |
|  | Beclomethasone - CpG double dose | >0.9999 |
|  | CpG single - CpG double dose | >0.9999 |
| I – III | Beclomethasone – CpG single dose | 0.7242 |
|  | Beclomethasone – CpG double dose | 0.9681 |
|  | CpG single – CpG double dose | >0.9999 |

| **Examination Stage** | **Treatments in Comparison** | **P-value (IL-17) BAL** |
| --- | --- | --- |
| I – II | Beclomethasone - CpG single dose | 0.0678 |
|  | Beclomethasone - CpG double dose | >0.9999 |
|  | CpG single - CpG double dose | >0.9999 |
| I – III | Beclomethasone – CpG single dose | 0.4953 |
|  | Beclomethasone – CpG double dose | 0.8391 |
|  | CpG single – CpG double dose | >0.9999 |

| **Examination Stage** | **Treatments in Comparison** | **P-value (IFNγ) BAL** |
| --- | --- | --- |
| I – II | Beclomethasone - CpG single dose | >0.9999 |
|  | Beclomethasone - CpG double dose | >0.9999 |
|  | CpG single - CpG double dose | >0.9999 |
| I – III | Beclomethasone – CpG single dose | 0.882 |
|  | Beclomethasone – CpG double dose | >0.9999 |
|  | CpG single – CpG double dose | >0.9999 |

| **Examination Stage** | **Treatments in Comparison** | **P-value (FoxP3) CD4 cells BAL** |
| --- | --- | --- |
| I – II | Beclomethasone - CpG single dose | 0.9012 |
|  | Beclomethasone - CpG double dose | >0.9999 |
|  | CpG single - CpG double dose | 0.6123 |
| I – III | Beclomethasone – CpG single dose | 0.5826 |
|  | Beclomethasone – CpG double dose | 0.1101 |
|  | CpG single – CpG double dose | 0.3783 |

| **Examination Stage** | **Treatments in Comparison** | **P-value (TGF-β) CD4 cells BAL** |
| --- | --- | --- |
| I – II | Beclomethasone - CpG single dose | 0.1209 |
|  | Beclomethasone - CpG double dose | 0.2853 |
|  | CpG single - CpG double dose | >0.9999 |
| I – III | Beclomethasone – CpG single dose | >0.9999 |
|  | Beclomethasone – CpG double dose | 0.4215 |
|  | CpG single – CpG double dose | 0.2595 |

| **Examination Stage** | **Treatments in Comparison** | **P-value (T-BET) CD4 cells BAL** |
| --- | --- | --- |
| I – II | Beclomethasone - CpG single dose | >0.9999 |
|  | Beclomethasone - CpG double dose | 0.5826 |
|  | CpG single - CpG double dose | 0.0258 |
| I – III | Beclomethasone – CpG single dose | >0.9999 |
|  | Beclomethasone – CpG double dose | >0.9999 |
|  | CpG single – CpG double dose | >0.9999 |

| **Examination Stage** | **Treatments in Comparison** | **P-value (GATA-3) CD4 cells BAL** |
| --- | --- | --- |
| I – II | Beclomethasone - CpG single dose | 0.8922 |
|  | Beclomethasone - CpG double dose | >0.9999 |
|  | CpG single - CpG double dose | 0.3816 |
| I – III | Beclomethasone – CpG single dose | >0.9999 |
|  | Beclomethasone – CpG double dose | 0.9189 |
|  | CpG single – CpG double dose | 0.7242 |

| **Examination Stage** | **Treatments in Comparison** | **P-value (IL-8) CD4 cells BAL** |
| --- | --- | --- |
| I – II | Beclomethasone - CpG single dose | >0.9999 |
|  | Beclomethasone - CpG double dose | 0.9996 |
|  | CpG single - CpG double dose | >0.9999 |
| I – III | Beclomethasone – CpG single dose | 0.7032 |
|  | Beclomethasone – CpG double dose | 0.6951 |
|  | CpG single – CpG double dose | 0.8871 |

## Effects of the Treatments on Clinical and Laboratory Parameters

**Table 4:** Comparison of the long-term effect sizes (I-III) of the three different treatment regimens from the current study (Beclomethasone, CpGsd, CpGdd), ordered according to parameter from the greatest to smallest effect

| **Beclomethasone I-III** | **Effect size** | **CpGsd I-III** | **Effect size** | **CpGdd I-III** | **Effect size** |
| --- | --- | --- | --- | --- | --- |
| Clinical scoring | 1.897 | HOARSI | 2.135 | Neutrophils | 1.232 |
| Nasal flaring | 1.670 | Nasal discharge | 1.787 | HOARSI | 1.189 |
| Breathing type | 0.967 | Clinical scoring | 1.757 | Nasal flaring | 1.107 |
| Nasal discharge | 0.901 | Breathing type | 1.670 | Clinical scoring | 1.085 |
| Auscultation | 0.710 | Nasal flaring | 1.538 | Breathing rate | 1.061 |
| HOARSI | 0.589 | Breathing rate | 1.334 | Viscosity | 1.043 |
| Neutrophils | 0.556 | Viscosity | 1.230 | AaDO2 | 1.030 |
| Interpleural pressure | 0.532 | Neutrophils | 1.064 | Nasal discharge | 0.909 |
| PaO2 | 0.514 | Auscultation | 0.973 | Tracheal mucus | 0.841 |
| Tracheal mucus | 0.483 | Tracheal mucus | 0.964 | Auscultation | 0.702 |
| AaDO2 | 0.441 | PaO2 | 0.856 | PaO2 | 0.697 |
| Viscosity | 0.200 | AaDO2 | 0.829 | Breathing type | 0.471 |
| Breathing rate | 0.161 | Interpleural pressure | 0.551 | Interpleural pressure | 0.186 |

## Comparison of the Effect Sizes to Previous Studies

In order to analyse the relevance of the number of inhalations, the effect sizes of the previous studies were compared with those of the current study. In the previous studies, the horses were treated five times (Klier et al. 2015) or seven times (Klier et al. 2018) with the single CpG dose.

**Table 5:** Comparison of the effect sizes of the CpGsd from the current study (10 inhalations) with two previous studies, in which the horses received 7 or 5 inhalation treatments, respectively. The results describe the effect between I and III examination, after a period of no treatment (with 10 inhalations, a period of 8 weeks with no treatment after the last inhalation; 7 inhalations, a period with 6 weeks; and 5 inhalations a period with 4 weeks without any treatment after the last CpG inhalation) to evaluate the long-term effect (of 8, 6 and 4 weeks, respectively).

| **10 Inhalations** | **Effect size** | **7 Inhalations** | **Effect size** | **5 Inhalations** | **Effect size** |
| --- | --- | --- | --- | --- | --- |
| HOARSI | 2.135 | HOARSI | n.s. | HOARSI | n.s. |
| Nasal discharge | 1.787 | Nasal discharge | 0.764 | Nasal discharge | 1.268 |
| Clinical scoring | 0.757 | Clinical scoring | n.s. | Clinical scoring | n.s. |
| Breathing type | 1.670 | Breathing type | 1.183 | Breathing type | 1.333 |
| Nasal flaring | 1.538 | Nasal flaring | n.s. | Nasal flaring | n.s. |
| Breathing rate | 1.334 | Breathing rate | 1.025 | Breathing rate | 0.341 |
| Viscosity | 1.230 | Viscosity | 0.615 | Viscosity | 1.177 |
| Neutrophils | 1.064 | Neutrophils | 0.119 | Neutrophils | 0.408 |
| Auscultation | 0.973 | Auscultation | 1.233 | Auscultation | 1.436 |
| Tracheal mucus | 0.964 | Tracheal mucus | 0.720 | Tracheal mucus | 1.456 |
| PaO2 | 0.856 | PaO2 | 0.739 | PaO2 | 0.224 |
| AaDO2 | 0.829 | AaDO2 | 0.588 | AaDO2 | 0.389 |
| Interpleural pressure | 0.551 | Interpleural pressure | 0.337 | Interpleural pressure | 0.291 |

**References**

1. Gerber, V., M. King, D. A. Schneider, and N. E. Robinson. 2000. Tracheobronchial mucus viscoelasticity during environmental challenge in horses with recurrent airway obstruction. Equine Vet. J. 32:411-417.
2. Robinson, N. E. 2001. International Workshop on Equine Chronic Airway Disease. Michigan State University 16-18 June 2000. Equine Vet. J. 33:5-19
3. Klier, J., S. Fuchs, A. May, U. Schillinger, C. Plank, and G. Winter, et al. 2012. A nebulized gelatin nanoparticle-based CpG formulation is effective in immunotherapy of allergic horses. Pharm Res. 29:1650-1657.
4. Klier, J., B. Lehmann, S. Fuchs, S. Reese, A. Hirschmann, and C. Coester, et al. 2015. Nanoparticulate CpG immunotherapy in RAO-affected horses: phase I and IIa study. J. Vet. Intern. Med. 29:286-293.
5. Grabner, A. 2005. Arterielle Blutgasanalyse. Pp. 429-430. in W. Kraft, and U. M. Dürr, eds. Klinische Labordiagnostik in der Tiermedizin. Schattauer, Stuttgart.
6. Robinson, N. E., M. A. Olszewski, D. Boehler, C. Berney, J. Hakala, C. Matson, and F. J. Derksen. 2000. Relationship between clinical signs and lung function in horses with recurrent airway obstruction (heaves) during a bronchodilator trial. Equine Vet. J. 32:393-400.
7. Gerber, V., R. Straub, E. Marti, J. Hauptman, C. Herholz, M. King, A. Imhof, L. Tahon, and N. E. Robinson. 2004. Endoscopic scoring of mucus quantity and quality: observer and horse variance and relationship to inflammation, mucus viscoelasticity and volume. Equine Vet. J. 36:576-582.
8. Deegen, E., and H.-J. Klein. 1987. Interpleuraldruckmessungen und Bronchospasmolysetests mit einem transportablen Ösophagusdruckmeßgerät beim Pferd. Pferdeheilkunde, 3:213-221
9. Gerber, V., H. C. Schott II, and N. E. Robinson. 2011. Owner assessment in judging the efficacy of airway disease treatment. Equine Vet. J.43:153-158.
10. Klier, J. 2011. Neuer Therapieansatz zur Behandlung der COB des Pferdes durch Immunstimulation von BAL-Zellen mit verschiedenen CpG-Klassen Thesis, LMU München.
11. VCOG, V. C. O. G.-. 2011. Veterinary cooperative oncology group - common terminology criteria for adverse events (VCOG-CTCAE) following chemotherapy or biological antineoplastic therapy in dogs and cats v1.1. Vet Comp Oncol.
12. Wärther, B. 2013. Nicht-viraler Gentransfer mittels Magnetofektion bei primären Zellen des Equinen Sarkoids. Thesis, Freie Universität Berlin.
13. Kleiber, C., B. C. McGorum, D. W. Horohov, R. S. Pirie, A. Zurbriggen, and R. Straub. 2005. Cytokine profiles of peripheral blood and airway CD4 and CD8 T lymphocytes in horses with recurrent airway obstruction. Vet. Immunol. Immunopathol. 104:91-97.
14. Chomczynski, P., and N. Sacchi. 1987. Single-step method of RNA isolation by acid guanidinium thiocyanate-phenol-chloroform extraction. Anal. Biochem. 162:156-159.
15. Horohov, D. W., R. E. Beadle, S.Mouch, and S. S. Pourciau. 2005. Temporal regulation of cytokine mRNA expression in equine recurrent airway obstruction. Vet Immunol Immunopathol. 108:237-245.
